# Decreased angiogenic and increased apoptotic activities of bone microvascular endothelial cells in patients with glucocorticoid-induced osteonecrosis of the femoral head

**DOI:** 10.1186/s12891-020-03225-1

**Published:** 2020-04-29

**Authors:** Huachen Yu, Pei Liu, Wei Zuo, Xiaowei Sun, Hongzhi Liu, Feifan Lu, Wanshou Guo, Qidong Zhang

**Affiliations:** 1 Department of Orthopaedic Surgery, The Second Affiliated Hospital and Yuying Children’s Hospital of Wenzhou Medical University, Wenzhou, China; 2 Graduate School of Peking Union Medical College, Beijing, China; 3 Department of Orthopaedic Surgery, China-Japan Friendship Hospital, Beijing, China; 4 Beijing University of Chinese Medicine, Beijing, China; 5 Peking University China-Japan Friendship Institute of Clinical Medicine, Beijing, China

**Keywords:** Bone microvascular endothelial cells, Angiogenesis, Apoptosis, Osteonecrosis of the femoral head, Glucocorticoids

## Abstract

**Background:**

Glucocorticoid-induced osteonecrosis of the femoral head (ONFH) is closely associated with the dysfunction of the bone microvascular endothelial cells (BMECs). The present study investigated the angiogenic and apoptotic activity of the BMECs in glucocorticoid-induced ONFH.

**Methods:**

This study enrolled a total of 12 patients, six of whom were assigned to the ONFH group whereas the other six served as the control group. The ONFH group was composed of patients with glucocorticoid-induced ONFH while the control group had femoral neck fractures. BMECs were isolated from the subchondral region of the femoral head. Cell proliferation, cell viability, tube formation assay, Transwell assay, TUNEL assay, and Western blot analysis were performed.

**Results:**

BMECs of the two groups were successfully isolated and identified. No significant differences were noticed in BMECs proliferation between the two groups. However, compared to the control, cell viability, tube formation, and migration of BMECs were significantly decreased and the number of TUNEL positive cells was markedly increased in the ONFH group. In the ONFH group, it was also noted that the amount of Bax and cleaved-caspase3 was elevated while that of Bcl-2 was reduced.

**Conclusion:**

The findings of our study revealed that BMECs obtained from the glucocorticoid-induced ONFH patients had decreased angiogenic and increased apoptotic activities, which could explain the pathogenesis and progression of glucocorticoid-induced ONFH.

## Background

Glucocorticoids are often used to treat diseases such as severe acute respiratory syndrome, organ transplantation, acute lymphoblastic leukemia, multiple myeloma, rheumatoid arthritis, and systemic lupus erythematosus [1–5]. Overdose usage of glucocorticoids is a proven cause of ONFH [6]. The ONFH is a disabling condition and often occurs in individuals between 20 and 50 years old [7]. Approximately 65% of ONFH patients eventually require total hip replacements, resulting in a substantial economic and medical burden to the patient and society [8]. Although the relationship between glucocorticoid administration and the development of glucocorticoid-induced ONFH is a well-known phenomenon, the mechanism of its pathogenesis remains obscure [9].

Recent reports have indicated that the femoral head microcirculation obstacle caused by a dysfunction of the bone microvascular endothelial cells (BMECs) could be significant in the development of glucocorticoid-induced ONFH [10, 11].BMECs line the sinusoids and inner layer of blood vessels and play a crucial role in vascular homeostasis and angiogenesis [12]. Also, the regulation of apoptosis during angiogenesis occurs in the BMECs [13]. It has been reported that the functioning of angiogenesis and blood vessel integrity is negatively related to the level of apoptosis [14]. Besides, several studies have reported that the dysfunction of regional endothelial cells due to continuous exposure to glucocorticoids can induce cell apoptosis and inhibit angiogenesis [15]. However, there are currently no reports on whether the angiogenic and apoptotic activity of BMECs is affected in glucocorticoid-induced ONFH patients.

This study postulated that the angiogenic and apoptotic activity of BMECs could be altered in patients with glucocorticoid-induced ONFH. This study tested the above hypothesis by investigating the angiogenic and apoptotic activities of BMECs isolated from patients with glucocorticoid-induced ONFH. A control group consisting of patients with femoral neck fractures was used.

## Methods

### Patients

The specimens were obtained from the Orthopedic Department of the hospital between March 2018 and November 2018. A total of 12 participants were enrolled, six of whom had glucocorticoid-induced ONFH. These six patients formed the ONFH group while the other six had femoral neck fractures, and these formed the control group. The inclusion criteria were (1) male or female patients between 40 and 70 years old; (2) diagnosis of glucocorticoid-induced ONFH or femoral neck fracture; (3) indications for total hip arthroplasty. The exclusion criteria included (1) alcohol-induced or trauma-induced ONFH and (2) preoperative diagnosis of either HIV, hepatitis B or C infections. The ONFH group comprised 3 women and 3 men (average age of 51.7 ± 5.2 years) at the time of surgery, while the control group comprised five women and one man (average age of 65.3 ± 2.9 years) at the time of surgery (Table 1). Femoral heads were removed during total hip arthroplasty, and a band saw was used to cut all the specimens into half at the coronary level. Half of the femoral heads were fixed in 10% formalin for examination while the remaining half was used for the isolation and culture of BMECs.

**Table 1:** Comparison of Patients in the ONFH and control group

Demographics	ONFH (*n* = 6)	Femoral neck fracture (*n* = 6)	*P*-value
Males	3	1	0.545
Females	3	5	
Mean ages(y)	51.7 ± 5.2	65.3 ± 2.9	< 0.01

### Hematoxylin-eosin (HE) staining

The collected tissues were fixed in 10% formalin after which they were decalcified in 10% EDTA solution for 6 weeks. The samples were subsequently dehydrated in a graded series of ethanol, embedded in paraffin, sliced into 5 μm thick sections. After staining with HE, a light microscope was used to examine the level of necrosis of the bone and marrow tissues. The existence of empty lacuna was used to indicate the level of osteonecrosis [16]. The average number of lacunae from three fields was calculated for each group.

### Isolation and culture of BMECs

The BMECs of patients undergoing hip arthroplasty were obtained as described previously [17]. Briefly, cancellous bones were harvested from the subchondral region of the femoral head. The bone debris was digested with 0.2% type I collagenase and 0.25% Trypsin–EDTA for 5 min. The reaction was stopped with the addition of Dulbecco’s modified Eagle’s medium (DMEM, Gibco, USA). The cell lysates were filtered with 70-μmol/L cell strainer and centrifugated at 1500 rpm for 6 min. The supernatant was removed and the cells were bathed in endothelial cell medium (ECM, ScienCell, USA) containing 5% fetal bovine serum (FBS), 5 ml recombinant human VEGF and antibiotics in a 37 °C humidified incubator with 5% CO_2_. A fresh medium was added the next day, and at intervals of 3 days subsequently. The cells were passaged when they reached 90% confluence.

### Immunofluorescence staining

Cells were embedded on round coverslips and allowed to adhere. They were fixed with 4% paraformaldehyde for 20 min, permeabilized with 0.1% Triton X-100 for 15 min and blocked with 10% FBS for 30 min at 37 °C. Then cells incubated with rabbit antibodies against CD31 (Abcam, 1:200) and vWF (Abcam, 1:200) overnight at 4 °C. Next day, Alexa Fluor™488 secondary antibodies (Invitrogen) were employed to immerse them for 1 h at 37 °C. Finally, slips were stained with DAPI for 30 s and analyzed with a fluorescence microscope.

### Examination of cell proliferation and viability

The proliferation of BMECs was assessed using the CCK-8 kit following the instructions on the kit. After BMCEs incubation in the 96 wells, 100 μL of ECM and 10 μL of CCK-8 solution were added to each well for a 2-h incubation period. The OD of each well was determined using a microplate reader at a wavelength of 450 nm.

The neutral red uptake assay was used to assess cell viability, as detailed before [18]. BMECs were added to 96-well plate (2 × 10^5^ cells/well) and allowed to adhere. Next, a fresh FBS-free medium was added and then the cells were treated with neutral red dye (in a fresh medium) for 2 h. Subsequently, a neutral red lysis buffer was used to remove the neutral red stain, and the OD was assessed at 570 nm.

### TUNEL assay

Apoptosis of BMECs was measured with TUNEL staining using the In Situ Cell Death Detection Kit (Roche). Cultured BMECs were collected in a six-well plate, 12 h post preparation. The cells were fixed with freshly prepared 4% paraformaldehyde for 1 h, incubated with 3% H_2_O_2_ and 0.1% Triton X-100 for 10 min, and washed thrice with phosphate-buffered saline (PBS) during each step. Cells were subsequently stained with DAPI according to the In Situ Cell Death Detection Kit manual. Images were observed and captured using a fluorescence microscope (Olympus, Tokyo, Japan). The number of apoptotic cells was quantified by counting TUNEL-positive cells by three independent researchers.

### Tube formation assay

A 96-well plate was coated with 100 μL of Matrigel (BD, USA) and then allowed to solidify and polymerize at 37 °C for 1 h. The Matrigel was subsequently overlaid with 100 μL of a suspension of BMECs (2 × 10^5^ cells/well). Tube formation by BMECs was observed under a microscope after 6 h of incubation and quantified by NIH Image J.

### Transwell assay

Corning transwell chambers (Corning, 8 μm, USA) were used for the transwell assay. For the migration assay, 5 × 10^5^ BMECs suspended in 200 μL serum-free medium were added in the upper chambers, and 500 μL ECM containing FBS were added in the lower chamber. Following incubation for 12 h, the upper chambers were washed with PBS, and the cells on the upper surface were wiped off using a cotton swab. Subsequently, cells on the bottom surface of the membrane were fixed in 4% paraformaldehyde and stained with 1% crystal violet. The number of migrated cells was assessed and counted under an optical microscope.

### Western blot analysis

Cellular total protein was extracted using RIPA lysis buffer and then quantified using the BCA kit (Beyotime, China). After that, proteins were denatured by heating at 95 °C for 5 min. A 30 μg sample of proteins were resolved by 10% SDS-PAGE and transferred to a PVFD membrane. The membrane was bathed in fat-free milk to block non-specific binding for 1 h followed by incubating overnight at 4 °C with primary antibodies against caspase-3 (Abcam, 1:1000), cleaved caspase-3 (Abcam, 1:1000), Bax (Abcam, 1:1000), Bcl-2 (Abcam,1:500), and β-actin (Abcam,1:3000). Subsequently, they were incubated with their corresponding secondary antibodies. The immunoblots were visualized with Electrochemiluminescence Plus Reagent (Invitrogen), and the expression was quantified with Image Lab 3.0 software.

### Statistical analysis

All data were expressed as the mean ± standard deviation (SD). Data analysis was done using one-way analysis of variance (ANOVA) and the Student’s t-test using SPSS version 21.0 (SPSS Inc., Chicago, IL, USA). A value of *P* < 0.05 was considered statistically significant.

## Results

### Clinical data and histological analysis

**Fig. 1:**
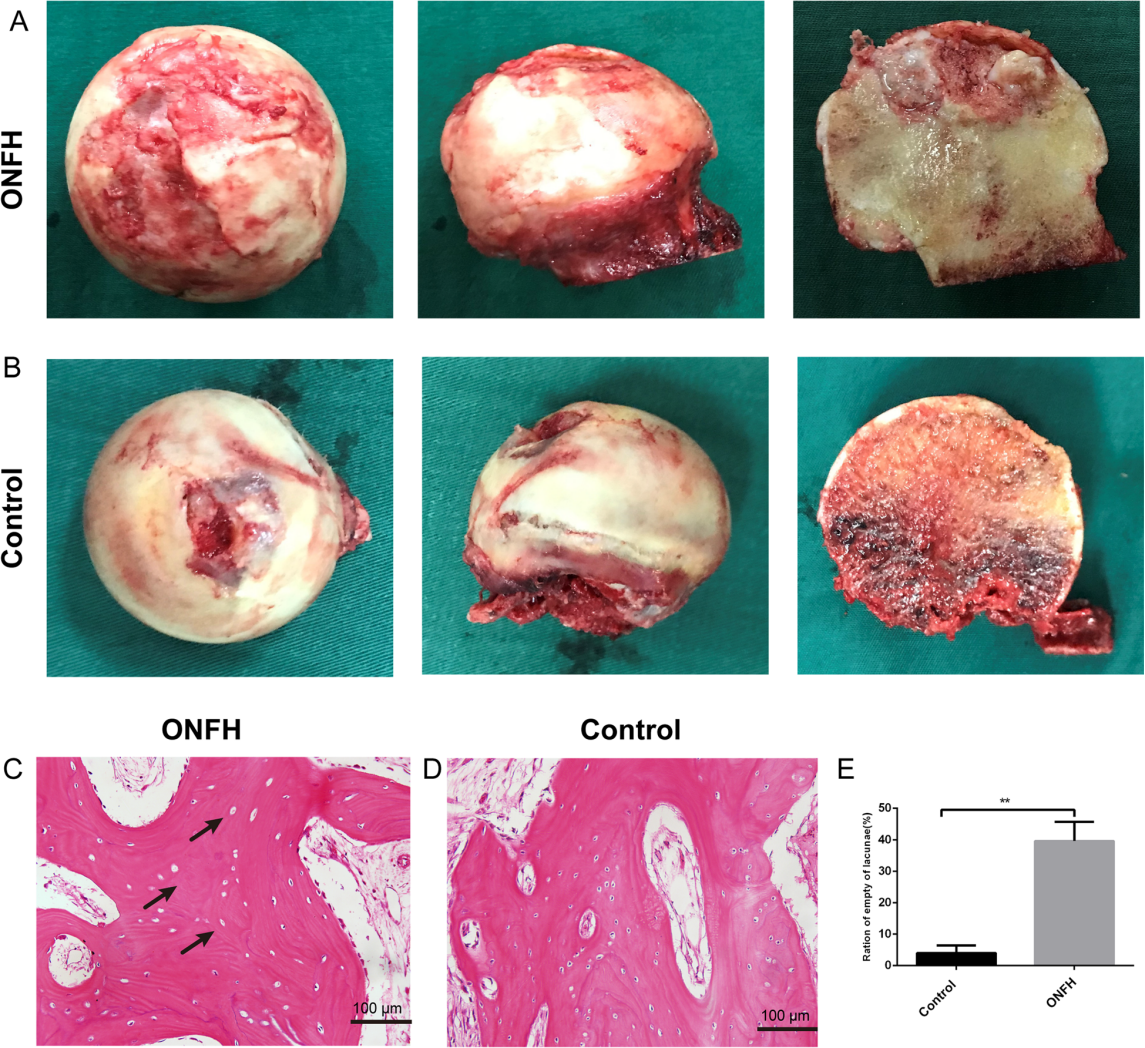
Clinical data of patients. Representative images of femoral heads from the ONFH (**a**) and the control group (**b**). Histological analysis of femoral heads (**c**-**e**). HE staining showing a disordered bone marrow structure and bone marrow necrosis, and empty bone lacunae found in the ONFH group. There was no ONFH in the control group. Bars depict the ratio of empty lacunae. Black arrows indicate empty lacunae (** indicates *P* < 0.01)

Representative femoral heads of the glucocorticoid-induced ONFH and the femoral neck fracture are indicated in Fig. 1a and b, respectively. Femoral head necrosis and slight bone marrow edema occurred in the ONFH group.

HE staining results showed that the bone marrow structure was disordered. Bone marrow necrosis and empty bone lacunae were observed in the ONFH group, while the control group had thick bone trabeculae and normal osteocytes (Fig. 1c-d). The proportion of empty bone lacunae was relatively higher in the ONFH group in comparison to the control group (*P* < 0.01), as shown in Fig. 1e.

### Cell culture, identification of BMECs

**Fig. 2:**
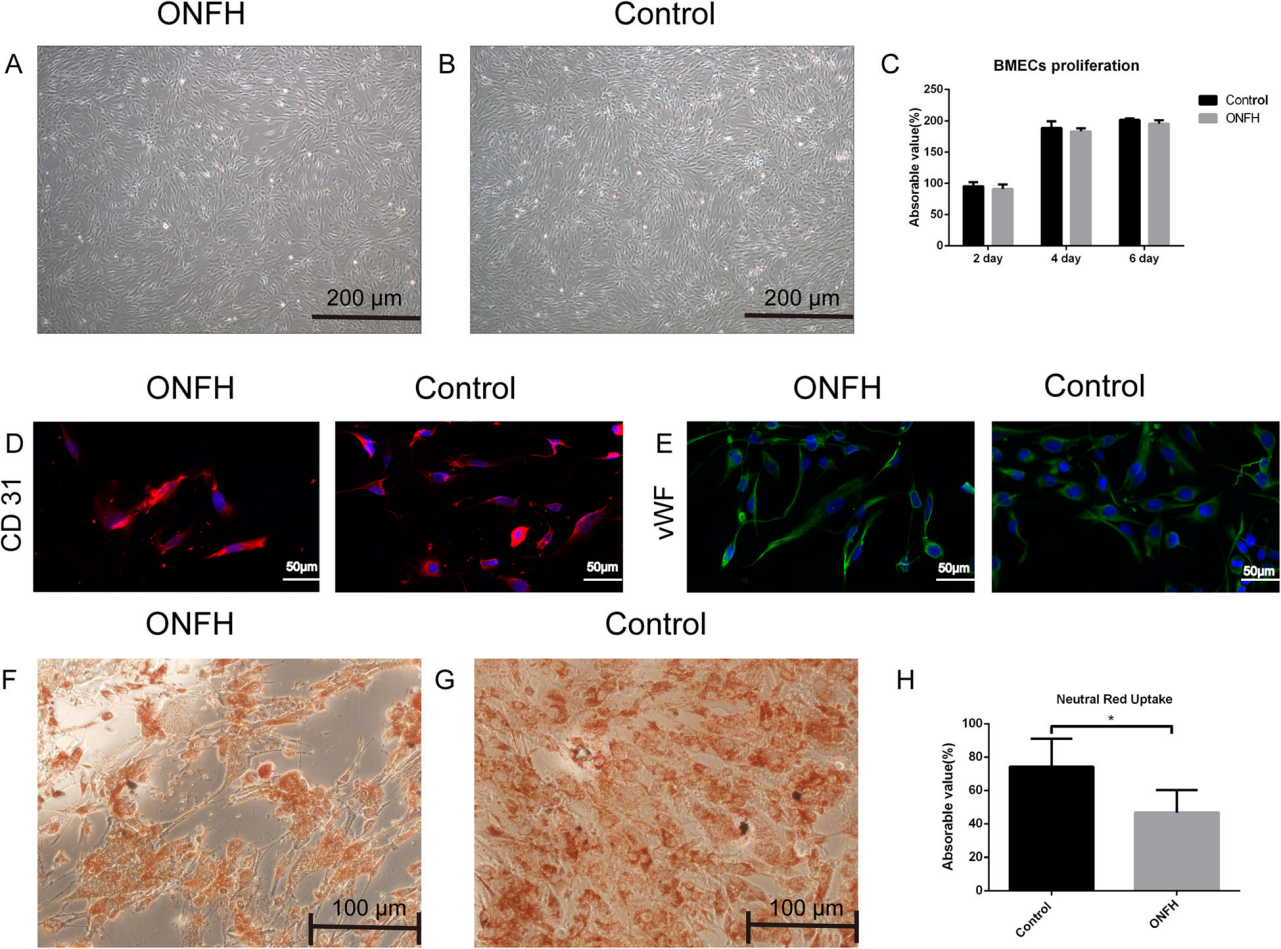
Comparison of morphology and growth potential of BMECs (at P2) from the group (**a**) and the control group (**b**). **c** Cell proliferation rates of BMECs in the ONFH and control groups. **d**-**e** Immunofluorescence staining results of CD31 and vWF in the ONFH and control groups. **f**-**h** Neutral red uptake assay showed fewer neutral red-stained cells in the ONFH group, but more red-stained cells in the control group (* indicates *P* < 0.05)

BMECs of the two groups were isolated from femoral bone tissue in the subchondral region. After 14 days of culture, the cells in the two groups developed the typical cobblestone morphology of endothelial cells. The morphologies of the P2 BMECs in the ONFH and control groups are shown in Fig. 2a and b, respectively. The isolated cells of the two groups showed a high expression of CD 31 and vWF (Fig.2d-e). These results indicated that these cells in the two groups were BMECs and thus were used in the following experiments.

### Cell proliferation and viability

Cell proliferation was evaluated 2, 4, and 6 days during the proliferation assay. No significant differences in cell proliferation were recorded between the two groups (*P* > 0.05; Fig. 2c). With regards to cell viability, however, more neutral red-stained cells were observed in the control groups than the ONFH group (*P* < 0.05; Fig. 2f-h).

### Decreased the angiogenic activity of BMECs in the ONFH group

**Fig. 3:**
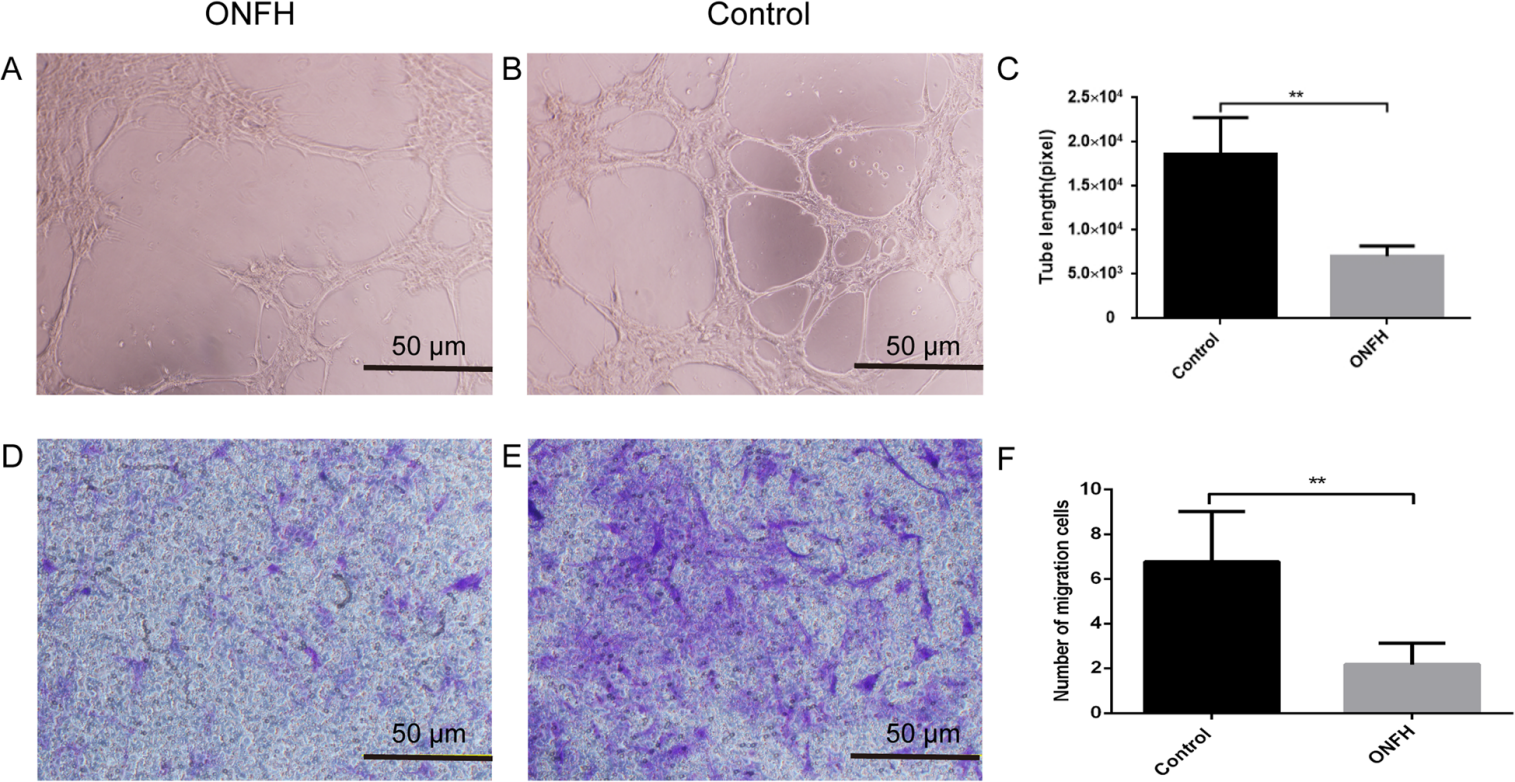
Angiogenic activity of BMECs. **a**-**b** Representative images of the tube formation assay from the ONFH group and the control group. BMECs from the ONFH group did not form tube-like structures on Matrigel, but control group BMECs successfully tube-like structure. **c** Quantification analysis of total tube length of BMECs (** indicates *P* < 0.01). **d**-**e** Typical results of the transwell assay of the ONFH group and the control group at 6 h are shown. BMECs from the ONFH group had a lower proportion of spreading cells (violet-stained) compared to the control group. **f** Quantification analysis of number migration cells (** indicates *P* < 0.01)

We used the tube formation and migration assays to compare the angiogenic activities of BMECs in the two groups. Whereas BMCEs in the control group formed elongated, vessel-like structures, the ONFH group BMECs formed incomplete or sparse tubular networks (*P* < 0.01 Fig. 3a-c). Migration capacity was remarkably decreased in the ONFH group relative to the control group (*P* < 0.01 Fig. 3d-f).

### Increased apoptosis activity of BMECs in the ONFH group

**Fig. 4:**
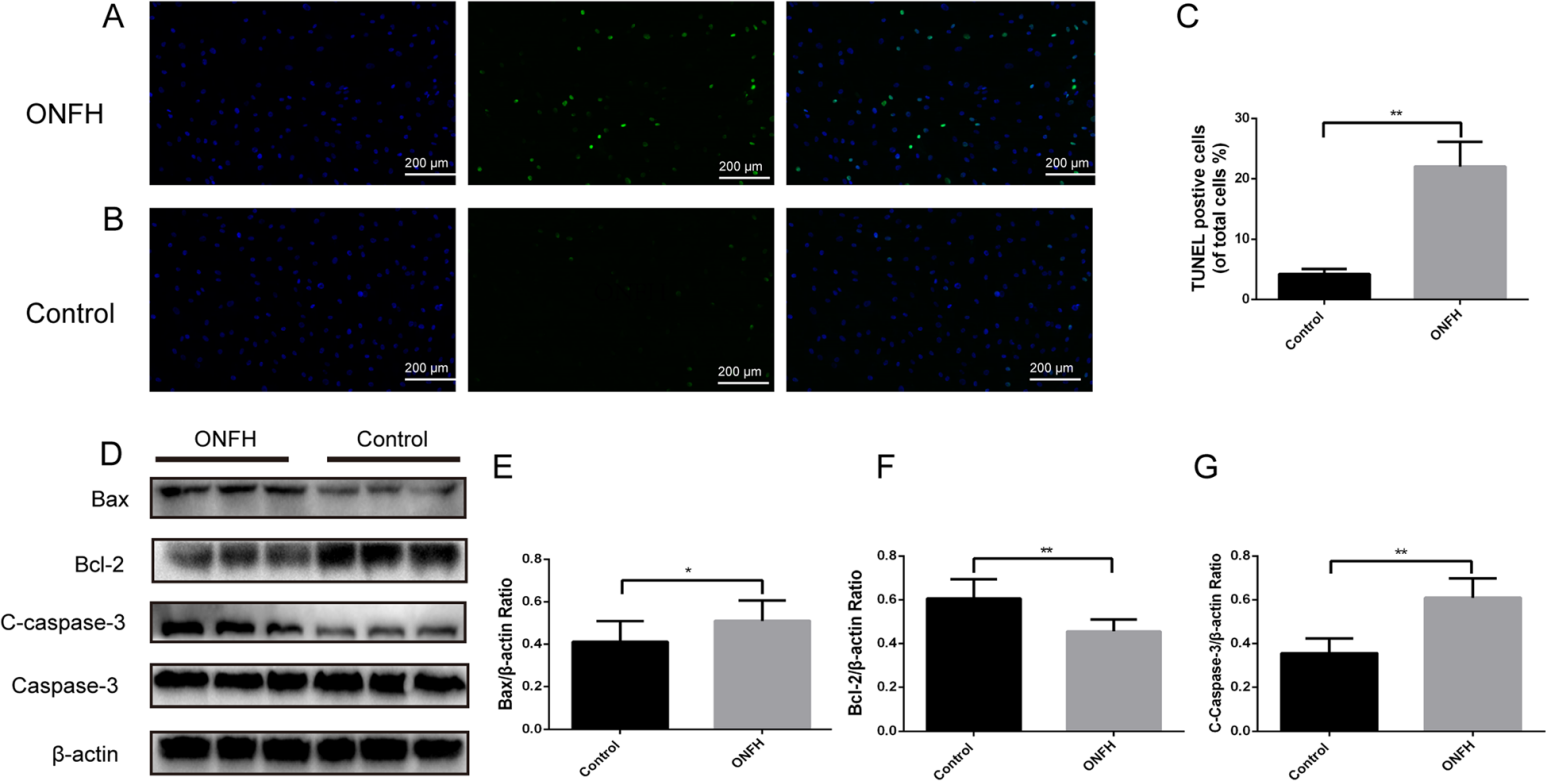
Apoptotic activity of BMECs. **a**-**c** Specimens from both groups were subjected to TUNEL assay. Analysis of the results indicated that TUNEL positive cells were markedly higher in ONFH groups in comparison with those in the control group (** indicates *P* < 0.01). Western blot and quantification analyses revealed the protein quantity of caspase-3, Bax, and Bcl-2 in BMECs of both groups. Each assay and analysis was run at least thrice (* indicates *P* < 0.05; ** indicates *P* < 0.01)

We first used the TUNEL assay to compare the apoptotic activities of the BMECs of the two groups. The TUNEL assay revealed that the apoptosis of BMECs was markedly higher in the ONFH group versus the control group (*P* < 0.05 Fig. 4a-c). Further attempts were made to assess the expression of proteins related to apoptosis such as cleaved-caspase 3, caspase 3,Bcl-2, and Bax. Our results showed that Bcl-2 was markedly decreased, while Bax was elevated in the ONFH group, relative to the control group. Moreover, it was noted that the expression levels of cleaved-caspase 3 were notably elevated in the ONFH group (*P* < 0.05 Fig. 4d-g).

## Discussion

Glucocorticoid-induced ONFH is an intractable and complex orthopedic disease caused by long term use or overdose of glucocorticoid treatment [9]. Studies have shown that the development of glucocorticoid-induced ONFH is closely associated with the dysfunction of BMECs [11]. This study reveals for the first time that BMECs obtained from patients with glucocorticoid-induced ONFH have decreased angiogenic and increased apoptotic activities compared with BMECs from patients with femoral neck fractures.

Although many theories regarding glucocorticoid-induced ONFH have been put forward, the underlying mechanism remains unclear. The dysfunction of BMECs has been reported to cause femoral head microcirculation obstacle, which is key to the development of glucocorticoid-induced ONFH [10]. Yu et al. [19] reported that glucocorticoids profoundly affect the transcriptome of BMECs during the early stages of glucocorticoid-induced ONFH. According to Yue et al. [18], glucocorticoids can induce BMECs to express various cytokines, which contributes to the development of glucocorticoid-induced ONFH. In the present investigation, we isolated BMECs from the subchondral region of the femoral head in the two groups. Cells in the two groups both formed the typical cobblestone morphology of endothelial cells and showed a high expression of CD 31 [20] and vWF [21], the standard markers of endothelial cells. These results indicated that these cells in the two groups were BMECs. Then, we used tube formation and migration assays to examine angiogenesis. The findings of this study indicate that the angiogenic activity of BMECs was profoundly impaired in the ONFH group in comparison with the control group. The reduced angiogenic activity of BMECs in ONFH patients may reflect the dysfunction of BMECs. It has proved that the dysfuntion of BMECs can impair vascular repair, blood vessel growth and finally decrease the local blood flow of the femoral head, which has been considered to be the cause of the glucocorticoid-induced ONFH [11]. Therefore, the reduced angiogenic activity of BMECs is thus a potential mechanism for glucocorticoid-induced ONFH.

In our study, patients in the control group, in which patients had femoral neck fractures, were significantly older than those in the ONFH group. The distinct features of the two conditions caused inevitable biases in the age of the two groups. Aging has been reported to be associated with endothelial dysfunction [22]. For instance, the study by Yavuz et al. [23] reported that endothelial function declines with an increase in the age of healthy human subjects. In our study, the angiogenesis of BMECs in the ONFH group was remarkably decreased as relative to the control group, even though the ONFH patients were much younger. This finding suggests that glucocorticoids accelerate the dysfunction of BMECs in ONFH patients.

Apoptosis is a normal physiological process that plays a crucial role in the pathogenesis and progression of glucocorticoid-induced ONFH [24]. Not only osteoblasts but also BMECs participate in the apoptosis process during the progression of ONFH. Kerachian et al. [25] pointed out that apoptosis of BMECs could activate the thrombosis cascade, followed by ischemia and infarction during the development of the glucocorticoid-induced ONFH. Also, Vogt et al. [26] showed that glucocorticoids could reduce angiogenesis by inducing the apoptosis of endothelial cells, and therefore injuring BMECs. Consistent with these studies, the TUNEL-positive cells in our study were only recorded in the ONFH group, suggesting that glucocorticoids induced apoptosis of BMECs in ONFH patients. It is known that caspase cascade plays an essential role in apoptosis and caspase-3 is activated during apoptosis [27]. Overexpression of caspase-3 has previously reported in ONFH patients [28]. Disruption of mitochondrial functions due to oxidative stress factors stimulates the caspase pathway, triggering apoptosis [29]. Also, the stimulation of cells by apoptotic factors facilitates dissociation of the original Bax and Bcl-2, causing an increase in Bax and decrease in Bcl-2 [30]. In this study, we demonstrated that Bcl-2 was markedly suppressed, while Bax and cleaved caspase 3 were increased in the ONFH group. These changes in expression levels suggest that the mitochondria participate in the apoptosis of BMECs in ONFH patients.

The following shortcoming should be underscored in this study. Firstly, the study was done in vitro and cannot, therefore, be replicated in the local environment of the femoral head, in the setting of glucocorticoid-induced ONFH. Consequently, the dysfunction of BMECs reported in this study may not hold for other etiologies of glucocorticoid-induced ONFH. Secondly, the sample size of our study was small and cannot, therefore, account for all patient comorbidities.

## Conclusion

In summary, the findings of our study indicate that BMECs obtained from glucocorticoid-induced ONFH patients have decreased angiogenic and increased apoptotic activities, which, to our knowledge, has not been previously reported. The decreased angiogenic and increased apoptotic activities may be responsible for the pathogenesis and progression of glucocorticoid-induced ONFH. Therefore, protecting the function of BMECs, such as promoting angiogenic or inhibiting apoptosis of BMECs, could offer an efficient therapeutic target for glucocorticoid-induced ONFH, which needs further exploration.

## Data Availability

The datasets used and/or analyzed during the current study are available from the corresponding author on reasonable request.

## Abbreviations

ONFH: Osteonecrosis of the femoral head
BMECs: Bone microvascular endothelial cells
HE: Hematoxylin-eosin
DMEM: Dulbecco’s modified Eagle’s medium
ECM: Endothelial cell medium
FBS: Fetal bovine serum
PBS: Phosphate-buffered saline
SD: Standard deviation
one-way ANOVA: One-way analysis of variance
